# The Role of Cyanidin-3-*O*-glucoside in Modulating Oxaliplatin Resistance by Reversing Mesenchymal Phenotype in Colorectal Cancer

**DOI:** 10.3390/nu15224705

**Published:** 2023-11-07

**Authors:** Hasan Kurter, Yasemin Basbinar, Hulya Ellidokuz, Gizem Calibasi-Kocal

**Affiliations:** 1 Department of Translational Oncology, Institute of Health Sciences, Dokuz Eylul University, Izmir 35330, Turkey; hasankurter1995@gmail.com; 2 Department of Translational Oncology, Institute of Oncology, Dokuz Eylul University, Izmir 35330, Turkey; ybaskin65@gmail.com; 3 Department of Preventive Oncology, Institute of Oncology, Dokuz Eylul University, Izmir 35330, Turkey; hulya.ellidokuz@deu.edu.tr

**Keywords:** oxaliplatin resistance, colorectal cancer, epithelial-mesenchymal transition, cyanidin-3-*O*-glucoside

## Abstract

Background: Epithelial-mesenchymal transition (EMT) plays an important role in the biological and biochemical processes of cells, and it is a critical process in the malignant transformation, and mobility of cancer. Additionally, EMT is one of the main mechanisms contributing to chemoresistance. Resistance to oxaliplatin (OXA) poses a momentous challenge in the chemotherapy of advanced colorectal cancer (CRC) patients, highlighting the need to reverse drug resistance and improve patient survival. In this study, we explored the response of cyanidin-3-*O*-glucoside (C3G), the most abundant anthocyanin in plants, on the mechanisms of drug resistance in cancer, with the purpose of overcoming acquired OXA resistance in CRC cell lines. Methods: We generated an acquired OXA-resistant cell line, named HCT-116-ROx, by gradually exposing parental HCT-116 cells to increasing concentrations of OXA. To characterize the resistance, we performed cytotoxicity assays and shape factor analyses. The apoptotic rate of both resistant and parental cells was determined using Hoechst 33342/Propidium Iodide (PI) fluorescence staining. Migration capacity was evaluated using a wound-healing assay. The mesenchymal phenotype was assessed through qRT-PCR and immunofluorescence staining, employing E-cadherin, N-cadherin, and Vimentin markers. Results: Resistance characterization announced decreased OXA sensitivity in resistant cells compared to parental cells. Moreover, the resistant cells exhibited a spindle cell morphology, indicative of the mesenchymal phenotype. Combined treatment of C3G and OXA resulted in an augmented apoptotic rate in the resistant cells. The migration capacity of resistant cells was higher than parental cells, while treatment with C3G decreased the migration rate of HCT-116-ROx cells. Analysis of EMT markers showed that HCT-116-ROx cells exhibited loss of the epithelial phenotype (E-cadherin) and gain of the mesenchymal phenotype (N-cadherin and Vimentin) compared to HCT-116 cells. However, treatment of resistant cells with C3G reversed the mesenchymal phenotype. Conclusion: The morphological observations of cells acquiring oxaliplatin resistance indicated the loss of the epithelial phenotype and the acquisition of the mesenchymal phenotype. These findings suggest that EMT may contribute to acquired OXA resistance in CRC. Furthermore, C3G decreased the mobility of resistant cells, and reversed the EMT process, indicating its potential to overcome acquired OXA resistance.

## 1. Introduction

The prevalence of CRC is such that it is the second most common type of cancer in women and the third in men [1]. The 5-year survival rate of primary and advanced metastatic diseases is 90% and 14%, respectively [2].

The therapeutic approach for localized CRC involves surgery and radiotherapy, whereas combination chemotherapy with 5-fluorouracil (5-FU) is the primary treatment for advanced CRC. This combination inhibits DNA and RNA synthesis, thereby limiting tumor growth [3,4,5].

Oxaliplatin, a platinum-based cytotoxic drug, binds to DNA and forms interchain adducts that induce transcriptional errors, leading to cellular apoptosis [6]. The combination of oxaliplatin with 5-FU has a 50% achievement drive in patients who have not received any prior chemotherapy treatment [2].

Unfortunately, chemotherapy failure often occurs due to drug resistance, which significantly contributes to poor prognosis [7].

There are multiple factors contributing to oxaliplatin (OXA) resistance. Theoretical mechanisms of resistance include the decreased expression of solid carrier membrane proteins, increased expression of multidrug resistance proteins, activation of the detoxification metabolism, activation of anti-apoptotic pathways, and activation of DNA repair mechanisms like nucleotide excision repair [8,9]. Additionally, epithelial-mesenchymal transition (EMT) induced by certain cytokines, such as TGF-β1 in the tumor microenvironment, can also lead to OXA resistance [10,11,12,13]. Therefore, comprehending the mechanisms underlying OXA resistance is crucial for successful CRC treatment.

EMT is a fundamental biochemical mechanism contained in tissue repair and embryonic development. It is characterized by a decrease in epithelial markers (occludin, claudins, E-cadherin, etc.) and an increase in mesenchymal markers (Vimentin, N-cadherin, etc.) [14,15]. Consequently, epithelial cells exhibit migratory and invasive behavior by losing their polarized phenotype, membrane adhesion, and cell-cell junctions. Cells undergoing EMT also acquire cancer stem cell properties and adopt a mesenchymal spindle-shaped phenotype [16,17], which may contribute to chemotherapy resistance [18,19].

In recent years, various studies have revealed the potential of secondary herbal metabolites, commonly found in traditional medicine, for cancer prevention and enhancing chemotherapy efficacy. These natural compounds, either used alone or in combination therapies, have shown inhibitory effects on proliferation, angiogenesis, and metastasis. Phytochemicals, which can be classified into two major classes based on their biosynthesis, namely phenolic and nitrogen-containing compounds, have been extensively investigated in this context. Among these compounds, anthocyanins (ACNs), which are water-soluble pigments, belong to the flavonoid-structured polyphenol group [20,21]. They are responsible for the characteristic colors such as red, purple and blue observed in various fruits [22]. Multiple studies in the literature have reported the health-promoting properties of ACNs, including their potential benefits against diabetes [23], cardiovascular diseases, neurodegenerative diseases [24], inflammation [25], and cancer [26].

The structural variations among anthocyanins primarily arise from the amount and location of attached sugars, the presence of aromatic or aliphatic acids linked to the sugar, the count of hydroxyl groups, and the positioning of these bonds [27]. The existence of hydroxyl groups in ACNs is associated with their antioxidant properties observed in food samples. Furthermore, the diversity of anthocyanins, which are glycosylated derivatives of anthocyanidins, varies depending on the specific anthocyanidin involved. The most abundant anthocyanidins existing in nature include malvidin, pelargonidin, delphinidin, peonidin, petunidin, and cyanidin [28].

C3G is the most abundant member of the ACN family [29], and its anticancer properties have been reported in colorectal [30], melanoma [31], and liver cancers [32]. For instance, Baster et al. demonstrated that C3G inhibits the formation of 3D spheroids [33]. Additionally, Jing et al. suggested that C3G can inhibit glucose transport and disrupt energy metabolism, leading to increased apoptosis in colorectal cancer through mitochondrial damage [34].

This study investigates, for the first time, the effect of C3G on acquired oxaliplatin (OXA) resistance in CRC cell lines. To evaluate this, we produced an OXA-resistant CRC cell line exhibiting a mesenchymal character. Our results reveal that targeting EMT biomarkers may be a viable therapeutic strategy for overcoming OXA resistance. Furthermore, we propose that C3G enhances the cytotoxicity of oxaliplatin and may serve as an effective treatment agent for overcoming acquired OXA resistance in CRC patients.

## 2. Materials and Methods

### 2.1. Cell Culture Condition

HCT-116 cell line (parental cell) was obtained from the American Type Cell Culture Collection. The HCT-116-ROx cell was generated by exposing parental cells to gradually increased concentrations of OXA over a period of six months. Both parental and resistant cells were grown in McCoy’s 5A culture medium (Cegrogen Biotech, E0500-250, Ebsdorfergrund, Germany) completed with 10% fetal bovine serum (Cegrogen Biotech, A0500-3030), 1% L-glutamine, and 1% penicillin-streptomycin antibiotics in a 5% CO_2_ atmosphere incubator at 37 °C. Additionally, culture medium of resistant cells was supplemented with 52 µM OXA (Sigma Aldrich, 61825-94-3, St. Louis, MO, USA).

### 2.2. Establishment of OXA-Resistant Cell

The HCT-116-ROx cell was developed over a period of six months by continuously increasing the concentration of OXA (1–52 µM). Initially, culture media supplemented with 1–10 µM OXA were used to prevent complete cell death at higher drug concentrations. Based on the proliferation and death rates of the cells, they were grown in a drug-free medium for 1–2 days. Subsequently, parental cells were treated with increasing concentrations of OXA for 2–3 days, followed by growth in a drug-free medium for 1–2 days. The development of drug resistance was confirmed when the proliferation rate of cells surpassed the death rate.

### 2.3. Determination of OXA-Induced Morphological Changes in Resistant Cells

To assess the morphological changes induced by OXA in resistant cells, brightfield images of the resistant cells were visualized by confocal microscope (Zeiss, LSM 800 Confocal, USA). The perimeter and area of both resistant and parental cells were measured using Zen 2.1 software (Zeiss, blue edition, San Diego, CA, USA). The shape factor of the cells was measured using the following formula, where A represents the area and P represents the perimeter [35]:

Shape Factor = 4πA/P2 (1)

### 2.4. Cytotoxicity Assay

The cytotoxic effects of OXA and C3G (Sigma Aldrich, 7084-24-4, USA) were evaluated using the WST-1 reagent, which measures the reduction of tetrazolium salts to formazan by mitochondrial dehydrogenase enzymes in viable cells. Cells were seeded in 96-well plates at a density of 1 × 10^4^ cells/100 µL per well and incubated for 24 h. OXA concentrations ranging from 3 to 192 µM were tested on both parental and resistant cells, while C3G concentrations ranging from 10–100 µM were tested on parental cells. After each treatment, 10 µL WST-1 reagent was added to each well and incubated at 37 °C for 4 h in the dark. Absorbance (ABS) was measured at 450 nm with a reference wavelength of 650 nm by the Varioskan Lux Microplate Reader (Thermo Fisher Scientific, VLBL00D2, Waltham, MA, USA). All experiments were performed in quadruplicate and repeated at least three times. Cell viability was measured via the following formula: Cell viability = (ABS _Drug_ × 100)/ABS _Control_. Additionally, the drug Resistance Index (RI), defined as the IC_50_ value of drug resistant cells divided by the IC_50_ value of parental cells, and the Reversion Index, calculated as the IC_50_ before reversion divided by the IC_50_ after reversion, were determined [36].

### 2.5. Hoechst 33342/PI Staining for Apoptosis Assay

Hoechst 33342/PI staining was employed to evaluate the impact of the combination of C3G and OXA on resistant cells. PI is a red fluorescent dye that stains nuclear and chromosomal DNA and is commonly used to detect late apoptotic and dead cells due to being impermeable to alive cells. Hoechst 33342 stains living cells in light blue and turns bright blue in apoptotic cells with condensed chromatin. Parental and resistant groups were seeded in a 96-well plate and treated with OXA (52 µM), C3G (20 µM), or a combination of OX + C3G (52 + 20 µM) for 48 h. Following the treatment, the cells were washed with PBS. A mixed solution containing 1 µg/mL Hoechst 33342 and 5 µg/mL PI was added to the cells and incubated for 30 min in the dark at 37 °C. Subsequently, the cells were washed with 1X PBS and imaged using a confocal microscope (Zeiss, LSM 800 Confocal, USA).

### 2.6. Wound-Healing Assay

To analyze the migratory capacity, HCT-116-ROx, HCT-116-ROx+C3G, and HCT-116 cells were grouped. HCT-116-ROx and HCT-116 cells were seeded in 6-well plates at of 3 × 10^4^ cells/2 mL. When the cell density reached 80–90%, a wound was created by scratching the cells using a 200 µL pipette tip. After washing with PBS, a group of HCT-116-ROx cells was treated with C3G (20 µM). All groups were monitored at 12, 24, and 48 h. The experiments were repeated at least three times. The migratory capacity of the cells was measured using the following equation:

Wound Distance % = (Wound distance h_0_ − Wound Distance h_x_)/Wound Distance h_0_ × 100 (2)

### 2.7. Immunofluorescence Staining

HCT-116-ROx and HCT-116 cells were seeded into Chambered Cell Culture Slides (Falcon, AZ, USA). A group of HCT-116-ROx cells was treated with C3G (20 µM) for 48 h. The cells were washed with 1X PBS and fixed with 4% paraformaldehyde for 20 min. After washing with 1X PBS, the cells were permeabilized with a 0.1% Triton X100 solution (diluted in 1X PBS) for 5 min at room temperature. For primary antibody staining, a staining buffer containing 1% BSA (diluted in 1X PBS) was prepared. The cells were then incubated overnight at 4 °C with primary antibodies against E-cadherin (1:250) (Abcam, Waltham, MA, USA), N-cadherin (1:500) (Abcam, USA), and vimentin (1:250) (Novus, St. Paul, MN, USA) in the staining buffer. Then, the cells were incubated with secondary antibodies, including Alexa Fluor-488-goat anti-mouse (1:1000) (Life Technologies, Carlsbad, CA, USA), Alexa Fluor-568 goat anti-rabbit (1:500) (Life Technologies, USA), and Alexa Fluor-647 goat anti-chicken (1:1000) (Jackson ImmunoResearch, Ely, UK) at room temperature for one hour. Subsequently, the cells were washed again with 1X PBS three times and incubated with 4′,6-Diamidino-2-Phenylindole Dihydrochloride (DAPI) (1:1000) (Invitrogen Molecular Probes, Eugene, OR, USA) for 10 min at room temperature to stain the cell nucleus. The stained cells were monitored under a confocal microscope (Zeiss, LSM 800 Confocal, USA), and fluorescence intensities were analyzed using Zen 2.1 software (Zeiss, blue edition, USA).

### 2.8. Quantitative Real-Time Polymerase Chain Reaction (qRT-PCR)

RNA isolation and cDNA synthesis were performed via the NucleoSpin RNA Kit (MN, 740955.50, Duren, Germany) and Evoscript Universal cDNA Synthesis Kit (ROCHE, 7912374001, Basel, Switzerland) following the manufacturer’s instructions, respectively. qRT-PCR was carried out using the FastStart Essential DNA SYBR Green Master Kit (ROCHE, 6402712001, Switzerland). The relative mRNA expression was normalized to GAPDH, serving as the housekeeping gene. The relative mRNA expression was calculated based on CT values using the 2^−(ΔΔCt)^ method.

### 2.9. Statistical Analysis

Statistical analysis was conducted using GraphPad Prism Software Version 8.4.3 (GraphPad Software-Inc., La Jolla, CA, USA). The data are presented as mean ± SD from at least three independent experiments, with each experiment performed in triplicate. The non-parametric Mann-Whitney U test was used to analyze the differences in data among the parental, resistant, and C3G-treated resistant cells. Statistical significance was denoted as *, **, and *** for *p* < 0.05, *p* < 0.001, and *p* < 0.0001, respectively.

## 3. Results

### 3.1. OXA Resistance Induces Spindle-Shaped Morphology in CRC Cells

**Figure 1:**
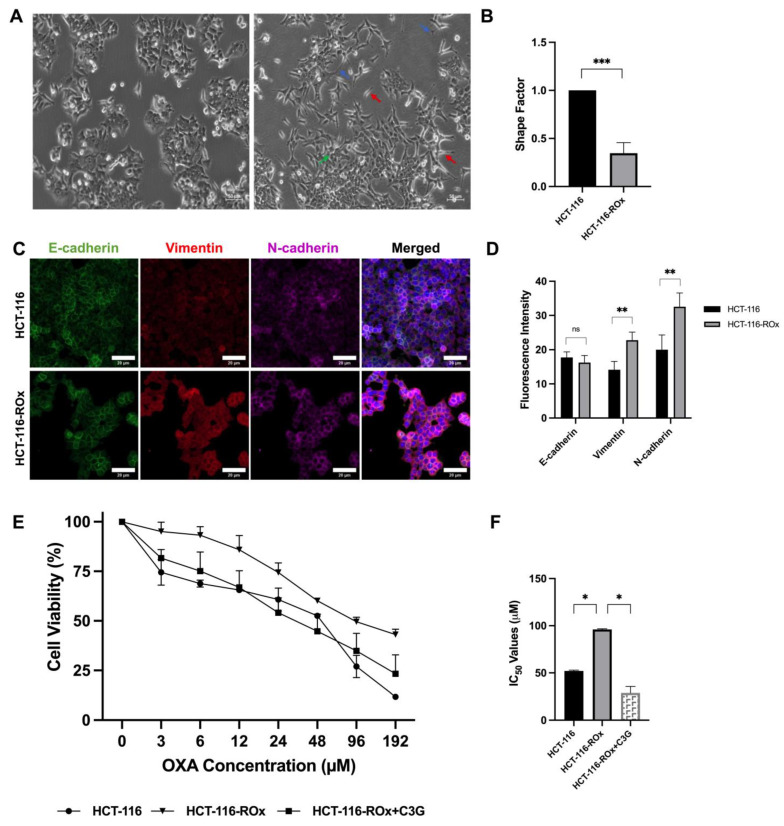
Comparison of parental and OXA-resistant CRC cells: (**A**) Morphological changes observed under light microscopy. The right and left images represent the parental and resistant cells, respectively (Scale bar 50 µm). OXA treatment induced distinct morphological alterations in HCT-116 cells. Blue, red, and green arrows indicate increased pseudopodia formation, spindle cell formation, and increased intercellular spacing, respectively. (**B**) Quantification of cell morphology using the shape factor, measured with ZEISS confocal microscopy (*** *p* < 0.0001). (**C**) Immunofluorescence staining of EMT markers in parental and resistant cells, visualized using ZEISS confocal microscope (Scale bar is 20 µm). (**D**) Analysis of fluorescence intensity in stained cells using ZEISS Software Version 2.6. The immunofluorescence staining assay was independently repeated at least three times. (**E**) Treatment of parental and resistant cells with different concentrations of OXA for 48 h. Higher IC_50_ values of OXA were observed in resistant cells compared to parental cells. The combination of C3G with OXA reversed the resistance in HCT-116-ROx cells. (**F**) IC_50_ values of parental, resistant, and combination treatment in resistant cells. All experiments were repeated at least three times. *, **, *** represent the *p* < 0.05, *p* < 0.001, and *p* < 0.0001, respectively.

The presence of adhesion proteins prevents the mobilization of epithelial cells. However, during the EMT process, cells undergo morphological changes from a round shape to a spindle-shaped morphology. These changes are attended by the loss of epithelial proteins and the gain of mesenchymal proteins [37]. In our study, the establishment of acquired OXA resistance led to significant morphological alterations in HCT-116-ROx cells, characterized by a loss of intercellular adhesion and an increase in spindle-shaped cells with the formation of pseudopodia (Figure 1A). These changes suggest the emergence of a mesenchymal phenotype associated with OXA resistance. The morphological changes in the resistant cells were quantified based on the ratio of cell area to perimeter, represented by the shape factor. The shape factor values for the resistant and parental cells were 0.347 and 1.0, respectively, indicating a significant difference (Figure 1B, *** *p* < 0.0001). Immunofluorescence staining for EMT markers revealed an increased expression of mesenchymal markers in the HCT-116-ROx compared to the HCT-116, further confirming the gain of a mesenchymal phenotype and the loss of cell interactions (Figure 1C,D, ** *p* < 0.001).

### 3.2. C3G Reverses OXA Resistance in HCT-116-ROx Cells

To investigate the mechanisms underlying OXA resistance in CRC, we obtained OXA-resistant cells by applying gradually increasing OXA to parental cells. Altered OXA sensitivity in resistant cells was assessed via WST-1. Cytotoxicity results showed that the sensitivity of resistant cells to OXA was significantly reduced compared to parental cells. The IC50 concentration of OXA was 52 µM in HCT-116 cells and increased to 96 µM in HCT-116-ROx cells (Figure 1E, * *p* < 0.05). The Resistance Index was derived from the IC50 concentrations of OXA for both the parent and resistant cells. For a 48-h incubation period with OXA, the Resistance Index between the resistant cells and their parental counterparts was determined to be 1.8 (Figure 1F, * *p* < 0.05). This value indicates that the resistant cells exhibit a resistance to OXA that is 1.8-fold greater than that of the parent cells.

Next, we examined whether C3G could reverse OXA resistance. Initially, the cytotoxic effects of C3G were assessed on parent cells, with results indicating no observable cytotoxicity (Supplementary Figure S1). Subsequently, based on these findings, a concentration of 20 μM was chosen for combined treatment due to its non-cytotoxic nature. HCT-116-ROx cells were treated with a combination of OXA and C3G. The data showed that C3G effectively reversed OXA resistance, as evidenced by a decreased IC_50_ value of 32.5 µM for the combination in resistant cells (Figure 1E, * *p* < 0.05). The Reversion Index was derived from the IC50 values associated with OXA and the combined treatment of OXA+C3G in HCT-116-ROx cells. The combined therapy revealed that C3G amplified the sensitivity of OXA by a factor of 2.95 in the resistant cells (Figure 1F, * *p* < 0.05). This finding indicates that C3G possesses potential efficacy in counteracting OXA resistance in HCT-116-ROx cells.

### 3.3. The Combination of C3G and OXA Increases the Apoptotic Rate in HCT-116-ROx Cells

To investigate the apoptotic effects of the C3G+OXA combination on HCT-116-ROx cells, Hoechst 33342/PI double staining was performed. Hoechst 33342 stains live cells with blue fluorescence, while PI specifically stains dead cells with red fluorescence that can penetrate the cell membrane.

**Figure 2:**
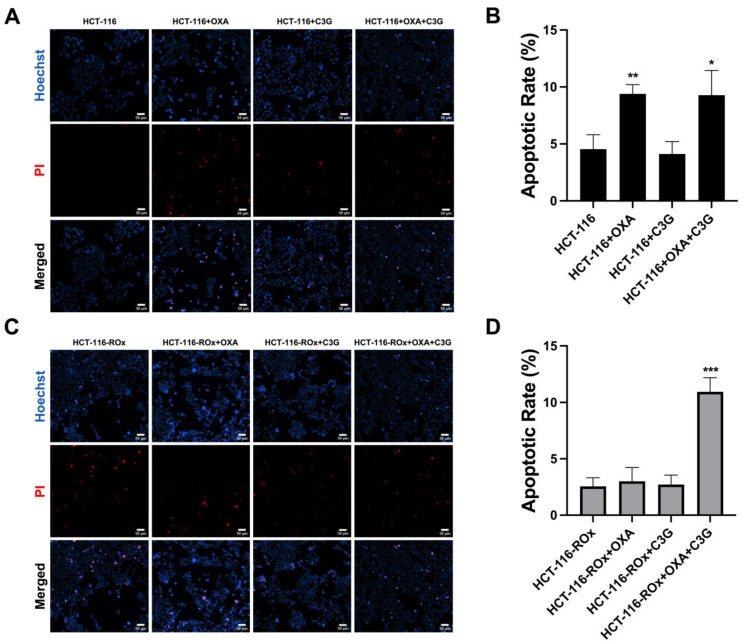
Hoechst 33342/PI dual staining analysis of parental and resistant CRC cells. (**A**–**C**) Fluorescence images of parental cells and (**B**–**D**) resistant cells. Living cells are indicated by light blue, while condensed cells are shown in bright blue. Red fluorescence represents apoptotic and necrotic cells (Scale bar is 50 µm). *, **, *** represent the *p* < 0.05, *p* < 0.001, and *p* < 0.0001, respectively.

In parental cells, OXA treatment increased the apoptotic rate compared to the control group (Figure 2A, ** *p* < 0.001). Similarly, the combination of C3G+OXA also increased the apoptotic rate compared to the control group, but C3G had no effect on the apoptotic phenotype. (Figure 2A, *p* > 0.05).

In our results, OXA (52 µM) and C3G (20 µM) had no effect on the apoptotic phenotype in resistant cells (Figure 2B, *p* > 0.05). Interestingly, however, there was a statistically significant increase in the apoptotic phenotype of the cells in combination of the agents (*** *p* < 0.0001).

These results suggest that the combination of C3G-OXA synergistically enhances the apoptotic response in HCT-116-ROx cells, indicating its potential for overcoming resistance and promoting apoptosis in resistant colorectal cancer cells.

### 3.4. C3G Attenuates the Increased Migration Rate in HCT-116-ROx Cells

To assess the effect of C3G on HCT-116 cell viability, we conducted viability assays. C3G did not significantly affect cell viability at concentrations of 10 and 20 µM after 48 h compared to the non-treated parental cells (Supplementary Figure S1, * *p* > 0.05). However, cell viability was significantly reduced at concentrations ranging from 30 to 100 µM compared to the parental cells (Supplementary Figure S1, * *p* < 0.05). Therefore, we selected 20 µM as the highest non-cytotoxic concentration of C3G for subsequent experiments.

**Figure 3:**
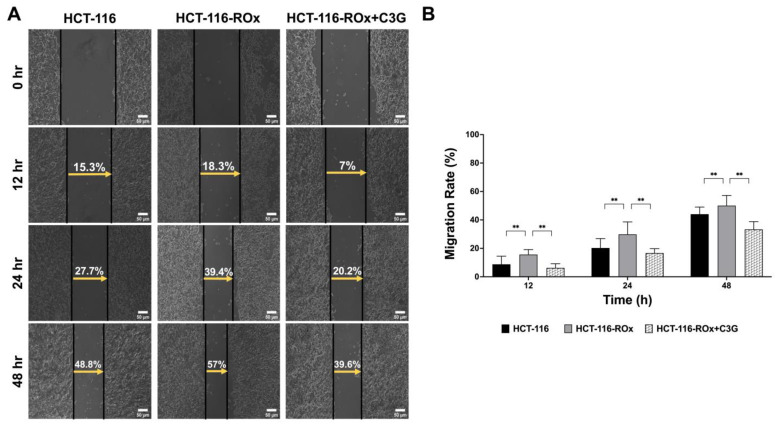
Migration capacity of parental and OXA-resistant CRC cells with cyanidin-3-O-glucoside treatment. (**A**) Wound-healing assay performed on parental, OXA-resistant, and C3G-treated OXA-resistant cells. Observations were made using an inverted microscope (ZEISS). (**B**) Migration distances were calculated using ZEISS Software Version 2.6. The wound-healing assay was independently repeated at least three times (Scale bar is 50 µm). ** represents the *p* < 0.001.

We evaluated the migration capacity of parental and resistant cells at the 12th, 24th, and 48th hours (Figure 3A). By the end of the 48th hour, the wound-healing distance covered by HCT-116-ROx cells was 57%, whereas HCT-116 cells covered 48.8% of the wound. The results of the migration assay clearly showed that the migration capacity of resistant cells was substantially higher than that of parental cells (Figure 3B, ** *p* < 0.001).

Furthermore, when OXA-resistant HCT-116-ROx cells were treated with C3G, the wound-healing distance decreased to 39.6% (Figure 3A,B ** *p* < 0.001). These findings indicate that C3G effectively inhibited the migration capacity of OXA-resistant CRC cells compared to non-treated HCT-116-ROx cells.

In conclusion, our results suggest that C3G has the potential to attenuate the increased migration rate observed in OXA-resistant HCT-116-ROx cells, highlighting its therapeutic potential in combating the migratory properties of resistant colorectal cancer cells.

### 3.5. C3G Reverses OXA Resistance-Induced Mesenchymal Phenotype in HCT-116-ROx Cells

**Figure 4:**
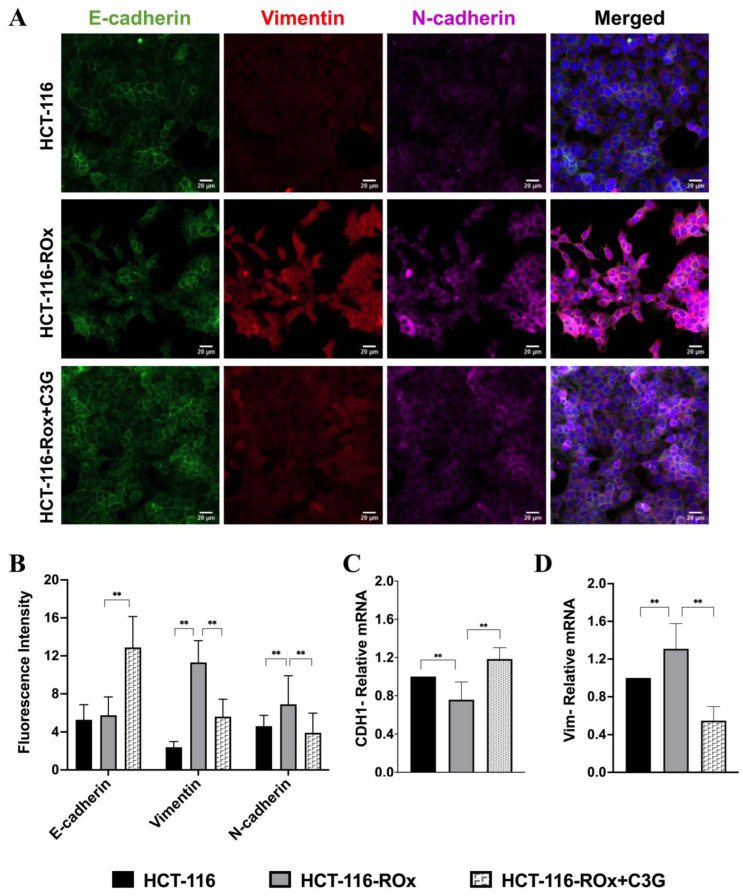
Expression of EMT markers was shown in parental and resistant cells with or without C3G treatment. (**A**,**B**) Immunofluorescence analysis of E-cadherin (green), vimentin (red), and N-cadherin (pink) in parental, OXA-resistant, and C3G-treated OXA-resistant cells using ZEISS confocal microscope. Fluorescence intensity analysis was performed using ZEISS Software Version 2.6. The immunofluorescence staining assay was independently repeated at least three times (Scale bar is 20 µm). (**C**,**D**) mRNA expression of E-cadherin and vimentin in parental, OXA-resistant, and C3G-treated OXA-resistant cells determined by qRT-PCR. qRT-PCR assay was independently repeated at least three times. ** represents the *p* < 0.001.

Fluorescence intensity analysis revealed a statistically significant increase in N-cadherin and vimentin expression in resistant cells compared to parental cells. (Figure 4A,B, ** *p* < 0.001). However, C3G-treated resistant cells showed an increased expression of E-cadherin and greatly decreased expression of N-cadherin and vimentin (Figure 4A,B, ** *p* < 0.001).

To further understand the molecular changes associated with EMT, we investigated the relative mRNA expression levels of EMT biomarkers in HCT-116 and HCT-116-ROx cells. We found that E-cadherin mRNA expression was decreased in resistant cells (Figure 4C, ** *p* < 0.05), while mRNA expression levels of N-cadherin and vimentin, markers of mesenchymal phenotype, were increased (Figure 4D, ** *p* < 0.05).

The qRT-PCR and immunofluorescence staining results showed consistent patterns, providing further evidence that the induction of EMT is responsible for the acquired OXA resistance in the CRC cell line. Moreover, C3G treatment effectively reversed the expression of the mesenchymal phenotype, indicating its potential in overcoming OXA resistance.

In summary, our findings suggest that C3G could reverse the OXA resistance-induced mesenchymal phenotype in HCT-116-ROx cells, as demonstrated by the downregulation of mesenchymal markers and the upregulation of epithelial biomarkers. This highlights the therapeutic potential of C3G in restoring the epithelial phenotype and overcoming OXA resistance in CRC cells.

## 4. Discussion

OXA, a key chemotherapeutic drug, is generally used in the treatment of advanced CRC. Its mechanism of action involves covalent linking to DNA, leading to the formation of guanine-guanine or guanine-adenine DNA adducts [38]. However, prolonged use of OXA often cause reduced drug sensitivity and the acquisition of drug resistance, posing a major challenge in CRC treatment [39]. Several processes have been proposed to contribute to OXA resistance, including increased an expression of ATP-binding cassette proteins, dysregulation of miRNAs, enhanced DNA damage repair mechanisms, and EMT [40]. For example, the multidrug resistance protein ABCG2 has been implicated in the development of OXA resistance in CRC [41], and the inhibition of miR-483-3p has been shown to confer OXA resistance in CRC cell lines [42]. Although important insights have been gained into the mechanisms underlying resistance to cisplatin, a first-generation platinum group, they are still poorly understood for OXA resistance [43]. Therefore, it is imperative to unravel the underlying mechanisms of OXA resistance and devise novel strategies to overcome it in CRC treatment.

Anthocyanins, classified as secondary metabolites in plants, exhibit potential anti-cancer properties by affecting various mechanisms. For instance, anthocyanins have the capability to scavenge free radicals and singlet oxygen by releasing hydrogen atoms from their phenolic rings [44]. It is crucial to note that reactive oxygen species can modulate cellular processes based on their concentration and duration of exposure, potentially leading to genomic instability and initiating various signaling cascades associated with carcinogenesis [45]. While elevated ROS levels can induce cytotoxic responses in cancer cells, modest ROS levels promote cell growth and survival through the activation of various pro-survival signaling mechanisms. Furthermore, studies have demonstrated that ROS can trigger EMT, a crucial process for kickstarting metastasis [46].

In this study, we generated a resistant strain by gradually applying OXA to parental cells. We investigated whether metastatic characteristics play a role in the development of OXA resistance in CRC. Additionally, we report for the first time that C3G can overcome OXA resistance by inhibiting cell migration and reversing the EMT process in colorectal cancer cells.

Overall, our findings clear up the mechanisms underlying OXA resistance and highlight the potential of C3G as a therapeutic strategy to overcome resistance and inhibit metastasis in CRC treatment. Further investigation of these mechanisms and evaluation of C3G in preclinical and clinical settings may offer promising avenues to improve the efficacy of OXA therapy in CRC patients.

The phenomenon of EMT is known to improve tumor invasion, metastasis, and drug resistance in various cancers. Consistent with previous findings, our study observed a higher migration ability in HCT-116-ROx compared to HCT-116, indicating the involvement of EMT [47]. Remarkably, we demonstrated that treatment with C3G decreased the migratory capacity of HCT-116-ROx cells. EMT has emerged as a robust mechanism in the gain of drug resistance across multiple malignancies [48]. TGFβ-induced EMT has been reported to confer drug resistance in colorectal cancer by promoting cellular plasticity [49]. Activation of the AKT/GSK-3β signaling pathway resulted in an increased expression level of Slug, an EMT transcription factor, resulting in decreased sensitivity to OXA [50]. Additionally, low dose oxaliplatin treatment has been shown to activate a mesenchymal phenotype in hepatocellular cancer cells [51]. Consistent with these observations, our study demonstrated that OXA treatment induced increased expression levels of mesenchymal proteins in parental cells. Moreover, administration of oxaliplatin in resistant cells further augmented the mesenchymal phenotype.

Taken together, these findings demonstrate the involvement of EMT in drug resistance formation and highlight the potential of targeting this process as a therapeutic approach. Our study provides novel insights into the interplay between OXA resistance and EMT in colorectal cancer, further emphasizing the potential of C3G in reversing drug resistance by modulating the mesenchymal phenotype. Continued research in this area holds promise for developing innovative strategies to get over drug resistance and improve the efficacy of CRC treatments.

Furthermore, EMT has been implicated in the modulation of apoptotic pathways. Zhang et al. proved that the development of drug resistance in CRC is associated with the activation of EMT and decrease in apoptotic proteins expression levels. However, the addition of curcumin to resistant cells reversed the EMT and increased the expression levels of pro-apoptotic proteins, suggesting a potential link between EMT and apoptosis regulation [37]. Similarly, Yin et al. reported that EMT was responsible for OXA resistance in CRC through the TGF-β/Smad2/3 pathway, which was associated with a decreased apoptotic rate in resistant cells. Interestingly, the combination of curcumin and OXA reversed drug resistance and EMT, leading to an increased apoptotic rate by enhancing caspase-3 activity [52].

In our study, we evaluated the apoptotic rate using Hoechst 33342/PI dual staining. Interestingly, there was no statistically significant difference in the apoptotic rate between the OXA-resistant CRC cell line and the non-treated resistant group. Although C3G alone did not alter the apoptotic rate, the combination of C3G with OXA significantly increased the apoptotic rate in resistant cells. These findings suggest that, while EMT and drug resistance may not directly influence the apoptotic rate in our model, a combination treatment with C3G and OXA can effectively enhance apoptosis in resistant cells, potentially through mechanisms beyond EMT modulation.

In summary, EMT has been linked to the regulation of apoptosis in drug resistant cells. Our research highlights the potential of combination treatments, such as C3G and OXA, to overcome drug resistance and increase the apoptotic rate, offering new strategies for improving therapeutic outcomes in CRC. Further investigations are warranted to elucidate the intricate interplay between EMT, drug resistance, and apoptosis, as well as to clarify the molecular mechanisms involved.

In our study, we examined the effect of EMT in the development of OXA resistance in CRC. As evidence, qRT-PCR and immunofluorescent staining methods showed that epithelial markers decreased, and mesenchymal markers increased in resistant cells. Interestingly, when HCT-116-ROx was treated with C3G, we observed a reversal of the EMT process. The ability of C3G to reverse EMT has also been reported in other studies. For example, Chen et al. showed that C3G inhibited EMT and suppressed the metastatic property of breast cancer [53]. However, our study is the first to show the effect of C3G on EMT, which is responsible for the formation of OXA resistance in colorectal cancer.

Taken together, our results suggest that EMT is an important process in drug resistance formation and that C3G, a natural compound, shows promise in reversing EMT in OXA resistance. This study gives some information to us about the molecular mechanisms of drug resistance and highlights the potential therapeutic application of C3G in combating resistance in CRC. Further studies are warranted to elucidate the specific mechanisms through which C3G exerts its effects on EMT reversal and its potential synergistic interactions with OXA in overcoming drug resistance.

## 5. Conclusions

In conclusion, overcoming drug resistance in colorectal cancer remains a significant challenge, particularly considering the complex interplay of intrinsic and acquired resistance mechanisms, as well as individual genetic and nutritional factors. It is crucial to deepen our understanding of the biological and biochemical processes underlying these resistance mechanisms. The improvement of current therapies and the development of new therapeutic agents are essential in tackling drug resistance effectively.

Our study contributes to this ongoing effort by demonstrating that C3G has the potential to overcome drug resistance in CRC by reversing the mesenchymal phenotype associated with resistance. By inhibiting the EMT process and its associated molecular changes, C3G shows promise as a natural compound for sensitizing resistant CRC cells to treatment. However, further research is necessary to fully elucidate the underlying mechanisms and evaluate the therapeutic potential of C3G in clinical settings.

Overall, our findings highlight the importance of exploring novel strategies to overcome drug resistance in CRC and provide a basis for future investigations aiming to enhance treatment outcomes and improve the prognosis for CRC patients.

## Data Availability

The data presented in this study are available in this article.

## Supplementary Materials

The following supporting information can be downloaded at: https://www.mdpi.com/article/10.3390/nu15224705/s1, Figure S1: The effect of C3G on HCT-116 cell viability was evaluated.
