# Supplementary figures and images for: The Role of Cyanidin-3-O-glucoside in Modulating Oxaliplatin Resistance by Reversing Mesenchymal Phenotype in Colorectal Cancer
Source: Nutrients. 2023 Nov 7;15(22):4705. doi: 10.3390/nu15224705 (PMC10674439; doi:10.3390/nu15224705)

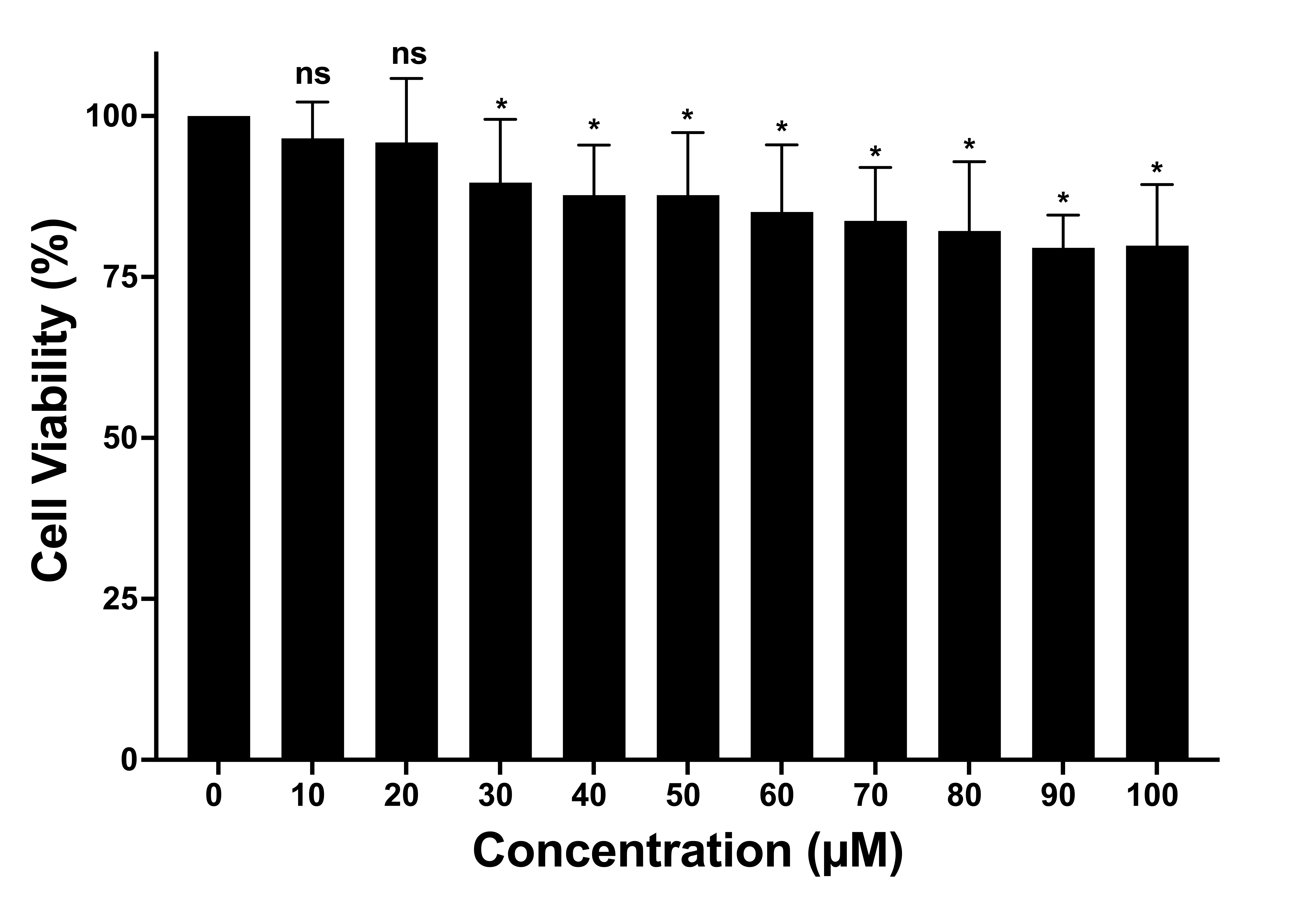

Supplement: Supplementary file 1 [file nutrients-15-04705-s001.zip › nutrients-2656830-supplementary.tiff]
